# The Hospital. Nursing Section

**Published:** 1902-06-21

**Authors:** 


### The Hospital.

### Nursing Section.

Contributions for this Section of "The Hospital" should be addressed to the Editor, "The Hospital" Nursing Section, 28 & 29 Southampton Street, Strand, London, W.C.

No. 821.—Vol. XXXII. *SATURDAY, JUNE 21, 1902.*

### Notes on News from the Nursing World.

### THE QUEEN AT ALDERSHOT.

Both the sisters at the Cambridge Hospital, Aldershot, and the nurses at the Louise Margaret Hospital had the gratification of welcoming Queen Alexandra on Sunday afternoon. The Queen, who was accompanied by the Prince and Princess of Wales, Princess Victoria, Prince Arthur of Connaught, Lord Roberts, and others, drove first to the Cambridge Hospital and proceeded direct to the reading-room, where the invalids from South Africa had been collected. Here her Majesty talked to some of them and inquired about their wounds and their present state of health, particularly a colour-sergeant, who is consumptive, and to whom the Queen gave some cough lozenges as she was distressed by his cough. The Royal party then visited several wards and conversed with the patients, asking many questions: being very interested in all they saw, and also in the hospital chapel where service is held every Sunday evening for the convalescent patients, and the kitchen where they inspected the sheet of diets for the day. The Royal party on leaving shook hands with the principal officials, including Superintendent Sister Grist, and her Majesty expressed herself extremely pleased with all she had seen, saying it was a beautiful hospital and extremely well managed; and conveyed the regrets of the King at not being able to be present, as he was laid up with lumbago.

### MENTIONED IN THE DESPATCH OF THE COMMANDER-IN-CHIEF.

In a despatch from Lord Roberts, published on Wednesday, and dated London, March 1st, 1902, the names of several nurses are mentioned as having rendered meritorious service. They were not, Lord Roberts explains, included before because the investigation into their claims for special mention was not sufficiently advanced, but the Commander-in-Chief requests that "all the mentions now made may be considered as bearing the same date—November 29th, 1900—as those in his despatch dated London, September 4th, 1901." The names of the nurses are as follows:—Mrs. Redpath and Mrs. Melina Rorke, Colonial Nurses, Buluwayo; Miss Knight, care of prisoners of war, Pretoria; Miss Frances Amelia Lowrie, matron, Bourke Hospital, Pretoria; Miss Ada Whiteman, matron, College Hospital, Maritzburg, and Leper Hospital, near Pretoria; Nursing Sister Miss Margaret Butler Alexander, Nursing Sister Miss Annie Henderson, Nursing Sister Miss Mary Ann Forbes, all of Queen Alexandra's Imperial Military Nursing Service Reserve, and the Scottish National Hospital; Mr. J. J. Reilly, and Mr. W. B. Ruth, male nurses, of the hospital ship *Maine*; Superintendent Miss Clara Mavesyn Chadwick, and Nursing Sister Miss Helen Hogarth, of the Queen Alexandra's Imperial Military Nursing Service Reserve, and the hospital ship *Princess of Wales*. Among other ladies who were also mentioned in the despatch are Lady Henry Bentinck, of the Portland Hospital; Lady Furley of the Red Cross Depot, Capetown; Mrs. Theodosia Bagot, of the Portland Hospital; Mrs. George Cornwallis-West, and Miss Eleanor Charlotte Warrender of the hospital ship *Maine*.

### NURSES AND THE CORONATION PROCESSIONS.

Many handsome compliments were paid in the pulpits of London churches on Sunday to hospital nurses. Possibly, some who heard and sympathised with these tributes may be glad of the opportunity of showing their sympathy in a practical manner. If so, we suggest that they should buy a few seats along the routes of the Coronation processions—there are plenty to be had at moderate prices on both sides of the Thames—and send them either to the matrons of the principal London hospitals, or to the Secretary of the Royal National Pension Fund for Nurses, 28 Finsbury Pavement, E.C., who will know how to distribute them. The sooner this is done the better, however, so that these nurses who already contemplate spending some of their hard-earned money on a seat, may be saved the expense of so doing. Speculators who have a number of places still unlet, might perhaps be glad to close with reasonable offers from purchasers who, in the kindness of their heart, preferred to hand over the seats to members of a class to whom it is admitted the public are heavily indebted.

### RESIGNATION OF THE MATRON OF ST. THOMAS'S HOSPITAL.

At the General Court of the Governors of St. Thomas's Hospital last week the retirement of Miss L. M. Gordon, the matron, was announced. The governors, having assented to resolutions of regret at her resignation, and of thanks to her for her past services, proceeded to declare the office vacant We understand that Miss Gordon is retiring altogether from hospital work, and, after 28 years' service, she has well earned a rest. She was trained on the Nightingale Foundation, and came back to London from Leeds Infirmary.

### A SEPARATIST MOVEMENT IN IRELAND.

A public meeting was held in Dublin last week for the purpose of promoting a movement for commemorating the Coronation by founding a fund for the benefit of aged, sick, or disabled certificated female nurses working in Ireland. Speeches were made on the occasion by the Lord Chief Justice of Ireland, Lord Justice Fitzgibbon, and others, who justly eulogised the excellent work which is being done by the nurses in the sister island. It was ultimately agreed to appeal to the public for support, though Sir Andrew Reed, by whom the enterprise was set on foot, admits that it is "somewhat indefinite." The point might be raised that if nurses exercised proper forethought there should be no need for it, and, by acting as a deterrent to thrift we think that it is far more likely to do harm than good. The distinguished men who dilated on the "impossibility" of nurses being able, under existing circumstances, to lay anything by for a rainy day, ignored the existence of the Royal National Pension Fund for Nurses, of which Queen Alexandra is President, notwithstanding that at the preliminary meeting Sir Andrew Reed alluded to it as a splendid institution. This fund is of course open to nurses in Ireland, and as a matter of fact many of them are already members. While, therefore, we are sure that the proposal to establish "King Edward the Seventh's Coronation National Fund for Nurses in Ireland" is well intended, we are of opinion that a far better project would be the creation of a fund by means of which nurses in Ireland, unable, on account of their small salaries, to pay the full premium to the Royal National Pension Fund, would receive assistance to the extent of 25 to 50 per cent. towards the payment of their own subscriptions. This, as well as encouraging thrift, would tend to cement the bonds between England and Ireland, whereas Sir Andrew Reed's project has the great disadvantage of being separatist in character. Why should the nurses in Ireland, who are to be called upon to subscribe to the new fund, with its necessarily heavy working expenses, separate themselves from their sisters in the United Kingdom and all parts of Greater Britain, and from the benefits which they, as the result of careful organisation, have obtained?

### ARRIVALS FROM SOUTH AFRICA.

The following Nursing Sisters have arrived from South Africa on board the *Nubia*, disembarking at Southampton on the 16th inst:—Acting Superintendent E. W. Gray (requires two months' leave, not returning to South Africa); J. M. Craig (requires one month's leave, not returning); A. L. Jones (requires one month, and returns); J. E. Whitehead (one month, not returning); L. Smith (one month and returning); E. J. Stonehouse (one month, returning). L. E. Jerram, Civil Nurse, requires one month's leave, and returns to South Africa. Invalids: L. Young, A.N.S.R. (three months' leave granted on medical certificate); B. Pocock, N.S.W. Nursing Staff (requires six weeks' leave and returns to South Africa.

### A QUEEN'S NURSE ON THE MIDWIVES' BOARD.

The Midwives Bill was read a third time in the House of Commons on Friday last. Mr. Renshaw expressed a hope that in the House of Lords a clause would be inserted providing that the Queen Victoria Jubilee Institute for Nurses should have a representative on the Central Board. Mr. Flower and Mr. T. P. O'Connor intimated their sympathy with the suggestion. Incidentally, Mr. O'Connor assured the House that nursing was at the root of the midwifery question. An effort to induce the Government to take charge of the Bill in the House of Lords was not successful, the Home Secretary rather pointedly remarking that the Peers were "beyond his control." But Lord Balfour of Burleigh may be relied upon to do all that can be done to add the measure to the Statute Book this year.

### "DURING THE PLEASURE OF THE COMMITTEE."

It is not probable that the pension scheme of the Governors of the London Hospital will create any enthusiasm among the nurses for whose benefit it is supposed to have been framed. The pension will be given after 18 years' service, and it will be continued "during the pleasure of the committee." There is thus no assurance of permanence, for whatever Mr. Sydney Holland and his colleagues may be able and willing to do while they are in office, they cannot bind their successors, and it may easily happen that it may be the pleasure of another committee that the pension should cease. This is such a serious drawback that, even if there be no difficulty about the funds which, it is stated, are to be administered out of the endowments of the hospital, we do not imagine any member of the nursing staff at the London will consider it prudent to rely for an income when she is past work upon the pleasure of a committee who may have the desire, as well as the power, to put an end to an arrangement so confessedly contingent upon circumstances.

### HOMES FOR PRIVATE NURSES.

A correspondent complains that there is no existing home for nurses in London at which they can live comfortably on terms within their income. Of the two she mentions, she says that living at St. Andrew's Home involves an expenditure of nearly £2 a week. This institution, however, has always been intended for the well-to-do few rather than for the many whose means are straitened. With regard to the hostel in Francis Street, although it may be true that the meal hours are not altered to suit the convenience of particular inmates, in one point at least our correspondent is, we think, mistaken. So far as the new south block—of which an account appears in another column—is concerned, no nurse need leave the home breakfastless. A penny placed in a slot of the gas-range on the landing will ensure the heating of water for tea, and the boiling of an egg. With a little forethought over night it should not be difficult to obtain a morning meal. In a large establishment, where economy has to be observed and service is an important question, it is unreasonable to expect meals at odd hours.

### TARDY REPARATION.

The Lambeth Guardians have at last repaired, as far as they could, the injustice done by them to Miss Mansell, the late superintendent nurse at Renfew Road Workhouse Infirmary. At the meeting last week Mrs. Despard very properly brought the question before the board, and appealed to them, "as an act of justice to Miss Mansell, to grant her a testimonial and to express the regret for the action they had taken." She was followed by Captain Andrew, who said that, "after the comments of Mr. Justice Grantham that the guardians did not go into the matter as he expected a public body would do, he thought it was incumbent upon the board to do justice to Miss Mansell." He added that "it was no little thing that the character of a blameless woman should have been so seriously attacked." We entirely agree with Captain Andrew, and we are glad that after these speeches, and a few words from the chairman, the guardians decided to grant the testimonial and to forward to Miss Mansell an expression of regret for the action taken by the board. But it is a pity that this course, instead of being pursued spontaneously, was not adopted until the Lambeth Guardians had been censured from the judicial bench.

### THE QUESTION OF STANDING.

Several correspondents have complained to us that a superfluous amount of standing is imposed upon nurses in hospitals, and we are disposed to think that here there is room for improvement. It is admitted by those who call attention to the question that a nurse must expect to be a great deal on her feet. In fact, it may be affirmed that unless a girl feels that she can bear a considerable amount of standing she should not decide to become a nurse. But the too rigid enforcement of rules as to standing cannot be defended even on grounds of expediency. The strain of being obliged to stand for many hours is distinctly calculated to accentuate, if not to produce physical weakness, and nurses suffering from physical debility not only end by becoming patients themselves, but are dulled in their mental activity by the weariness of prolonged standing on tired and aching feet. We hope that medical men, whom we believe are wrongfully accused of apathy in the matter, will exert their influence, wherever they can, to secure alleviations of a great evil.

### CANCER HOSPITAL, BROMPTON.

At the examination recently held at the Cancer Hospital, Fulham Road, on the termination of the lectures given by the medical staff during the winter session, the following probationers secured the first three places and will be awarded the three prizes offered by the Committee of Management of the hospital:—Nurses A. Wood, A. Dow, and D. M. Pinser.

### A HINT FROM HARTLEPOOL.

It was announced at the annual meeting of the West Hartlepool Nursing Association that as the result of addresses delivered to the employés at local works, the men had agreed to subscribe. We are convinced that, as a rule, appeals of this kind are not without effect, and it is a pity that the friends of nursing associations so frequently fail to tap a source of support which lies close at hand. The Hartlepool Association is in a highly satisfactory position from every point of view. Last year the nurses paid 8,075 visits as against 7,506 in the previous year; the number of cases nursed being 348 as against 261. Financially, a decrease in subscriptions was almost balanced by an increase in donations, and the credit balance is £354. There is no better way of keeping up the balance than by inducing the working classes to render systematic aid.

### BUNGLING AT BISHOP AUCKLAND.

The unfortunate action of the Bishop Auckland Nursing Association in refusing to elect any of the doctors of the town on the executive committee and the consequent determination of the doctors not to employ the nurses of the Association, have produced most untoward results. At a meeting of the subscribers, called together by the committee, owing to the continued lack of funds, the question whether it was worth while to maintain the organisation was discussed. It was stated that the nurses had practically no work to do, that the Association was in debt about £65, with £15 a month expenses to face, and a debt on the building. We should have imagined that the remedy for this condition of affairs was an amicable understanding with the doctors. But though Dr. Wardle attended the meeting and did his best to throw oil on the troubled waters, the only decision arrived at was to retain the nurses until the next annual meeting and then run the Association "on a provident basis." The Vicar of Auckland thinks that there are not enough sick poor in the town to need the Association exclusively; but if it asks for payment for the nurses, it will cease to be connected with Queen Victoria's Jubilee Institute.

### A HOME FOR COCKERMOUTH.

The generosity of Mr. Thomas Williamson, of Oakhurst, Cockermouth in presenting a nursing home to the town has had a stimulating effect. Mr. Williamson has not only given the house and premises for the purpose, but has also contributed £50 towards the cost of furnishing the home, and £25 for three years. In order to render the institution of real practical use, the income of the nursing association requires to be raised to the extent of about £130 a year, and at a meeting last week several substantial donations and subscriptions were announced.

### QUEEN'S NURSES IN IRELAND.

It is not only in the remote island of Achill that the services of a Queen's nurse are appreciated. From Portadown in county Armagh comes a striking evidence of the manner in which the work carried on in that town is regarded. "The time," says the report, which was read at the annual meeting of the Nursing Society, "is fortunately passed when it might have been necessary for the report to take up the burden of convincing the people of Portadown of the usefulness of the work done by the society, and the justice of the claim for the support of the public." The number of visits paid by the nurse last year was 5,067, an increase of 36 on the previous year.

### AN EXAMPLE TO COLLECTORS.

At the annual meeting of subscribers to the Maryhill District Nursing Association it was stated that the number of subscriptions obtained by single collectors was "more surprising than ever." One lady collected no fewer than 311 subscriptions, another 297, two others more than 200, and four upwards of 100 each. The subscribers to the association now number 2,252, an increase of 187 over those of the previous year, and though the expenditure was larger the income has more than kept pace with it. This is an admirable object lesson as showing what can be achieved by the enthusiasm and persistence of individuals.

### SHORT ITEMS.

The nurses of the Royal Infirmary, Newcastle-on-Tyne are to be presented on Saturday with a piano for their home in Ravensworth Terrace.—The sum of £1,340 16s. 9d. has been presented by Viscountess Downe as the contributions collected by her in the North Riding of Yorkshire towards the Women's Memorial to Queen Victoria.—The matron of Frame Food have published a little pamphlet entitled "Bringing up Baby, by a Hospital Nurse," in which many directions are given as to how to deal with the infant, the leading idea being the use of a certain proportion of Frame Food in its diet.

### Lectures on Gynæcology for Nurses.

By Robert Jardine, M.D., M.R.C.S., F.F.P. and S.G., F.R.S.E., Senior Physician to the Glasgow Maternity Hospital, Examiner in Midwifery to the University of Glasgow.

### LECTURE XIII.—DISPLACEMENT OF THE UTERUS.

As the uterus is a movable organ, it is liable to be displaced in various directions. It is also liable to changes in its natural curvature by bends or flexions. These are usually classed as displacements, though strictly speaking they are not true displacements.

The uterus, as you know, lies in the cavity of the pelvis, slung there by its various ligaments. Below it rests on the floor of the pelvis, its neck piercing the anterior vaginal wall. In front we have the bladder and behind the rectum. When the bladder is full the uterus is pushed backwards and somewhat upwards, and if the urine is evacuated no harm results, as the uterus sinks into its normal position again, but if the bladder is allowed to become over distended, the uterus will probably be tilted so far back that the fundus topples backwards, and you get a posterior displacement. In bad constipation the overloaded bowel presses the uterus downwards and forwards and tends to displace it. The abdominal pressure constantly acts upon the uterus, and would displace it downwards if it were not supported by the pelvic floor. If the interabdominal pressure is greatly increased as in severe straining at stool or in vomiting the uterus will be forced down. If the pelvic floor is weakened in any way, the downward displacement is liable to continue. The normal position of the uterus is in the pelvic cavity curved forwards against the bladder.

*Anteflexion.*—By a flexion of the uterus we mean that instead of being curved it is acutely bent. Anteflexion means an acute bending forwards, which is an exaggeration of the normal condition. The bend is at the junction of the cervix with the body of the uterus. It is sometimes a congenital condition, the whole uterus being imperfectly developed. In such a case the cervix will be small and the os pin-hole looking downwards and forwards. The acquired form is due to shortening of the posterior or utero-sacral ligaments from contraction after cellulitis in these ligaments. This drags back the uterus at the junction of the cervix and body, and thus causes an acute bend. A small fibroid tumour in the anterior wall near the fundus is liable to cause bending by its weight. In the early months of pregnancy the anterior bending of the uterus is exaggerated by its own weight.

*Symptoms.*—The patient, as a rule, suffers severe pain at her menstrual periods, and she will probably be sterile. The menstrual flow is sometimes increased. Sexual intercourse will usually cause pain. Leucorrhœa is apt to be present. She may be troubled with frequency of micturition from the uterus pressing upon the bladder and irritating it. This is generally seen in the early months of pregnancy.

*Diagnosis.*—The cervix is usually high up, the os small and looking downwards and forwards. A rounded body, the fundus, will be felt in the anterior fornix continuous with the cervix, but bent at an acute angle. The contracted utero-sacral ligaments may be felt through the posterior fornix.

*Prognosis.*—It is a difficult condition to cure, so the prognosis must be guarded.

*Treatment.*—If there is any inflammation present, this must be treated before an attempt is made to straighten the uterine axis. Uterine stem pessaries are sometimes used. The uterus is straightened, and the stem of the pessary is inserted into the cavity and kept in position by resting on a vaginal pessary. It is a somewhat dangerous instrument for a patient to wear, and is now seldom used. Different forms of vaginal pessaries have been invented, but none of them are of much use. Several operations have been devised to straighten the canal by cutting through the posterior lip of the cervix. If the woman should have a child the condition will be cured.

*Retroversion and Retroflexion.*—These are posterior displacements of the uterus. In retroversion the uterus is displaced backwards, but there is not an acute bend in it as in retroflexion. We shall consider the two together. Every time the bladder becomes very full the fundus of the uterus is raised and pushed back, but when the bladder is emptied it sinks forwards into its normal position. A sudden strain or blow when the bladder is full may easily cause the uterus to fall backwards. After an attack of inflammation the adhesions formed behind the uterus as they contract will drag it backwards. Backward displacement is common during the puerperium while the uterus is heavy. In the first stage of prolapse, or falling of the womb, there is some backward displacement. In the early weeks of pregnancy we often get the same condition, as the weight of the growing uterus makes it sink into the pelvis and the fundus passes slightly backwards. This may become exaggerated and end in retro-version or flexion of the pregnant uterus and give rise to a very serious condition, as the growing uterus will force the cervix against the neck of the bladder and cause retention of urine.

*Symptoms.*—The patient usually complains of weakness in the back and more or less pain, especially at her periods, which will probably be profuse and frequent. She may also suffer from leucorrhœa and sterility, and if she becomes pregnant an abortion will be liable to occur.

*Diagnosis.*—A vaginal examination will reveal that the cervix is low down in the pelvis, and the os looks downwards and forwards in retroversion, and directly downwards in retroflexion. A body will be felt in the posterior fornix. If it is a flexion the body will be low down, and the angle of its junction with the cervix will be easily made out. If it is a version, the posterior wall will be straight and not bent. If the surgeon passes a sound it will go backwards. A fibroid in the posterior wall of the uterus will feel very like a retroflexion, but by a careful bimanual examination, one may be able to make out the body of the uterus as well as the fibroid, and the sound will pass forwards. The uterus is usually enlarged. The sound must never be used until the possibility of pregnancy is excluded. The condition must be differentiated from fæces in the rectum, any deposit in the pouch of Douglas such as from cellulitis peritonitis, hæmatocele, cancer, an enlarged or cystic ovary and a fibroid of the posterior wall of the uterus, etc. A careful consideration of the history, and a judicious use of the sound will usually enable the surgeon to make a correct diagnosis.

*Prognosis.*—The chances of cure depend upon the amoun of fixation of the uterus by adhesions.

*Treatment.*—Inflammatory conditions must first be relieved by appropriate treatment, such as douching and glycerine and ichthyol tampons, etc. If the uterus is quite movable, it may be replaced at once. This is sometimes done bimanually by pressing the fundus up with the inside fingers, and then tilting it forwards, with the hand on the abdomen. The best way to press it up is by passing the middle finger of the internal hand into the rectum and the forefinger into the vagina. The rectal finger gives good leverage on the fundus, while with the forefinger the cervix can be pushed back. Another method of replacing is by means of the sound. When the uterus is replaced it should be kept in position by a suitable Hodge or Albert Smith pessary. The upper bar of the pessary lies behind the cervix when it is in its proper position, and the lower part lies in the vagina—not resting on the top of the symphysis. The pessary is passed first in the long axis of the vulva and is then rotated half-way round. The upper bar will invariably be found to lie in front of the cervix, and it must be brought behind the cervix by passing a finger in and hooking it back. A well-fitting pessary should not cause the patient any pain. In fact, she should not be aware of its presence. It should never be left in longer than three months at the most without being removed and cleaned. If it is left in for a long time it will become coated with a crust of phosphatic deposit, and may set up ulceration of the vagina and cause an opening into the bladder or rectum, or both. The patient should douche occasionally. In cases where there are adhesions it may be possible to gradually stretch them by tampons until the uterus can be replaced. They may be forcibly stretched or torn through, but this is apt to set up inflammation.

When the uterus is enlarged, and endometritis is present, curetting is necessary as well as replacement, and if the perineum is torn it should be repaired. For cases in which a pessary fails to keep the uterus in position an operation may be done. The round ligaments may be shortened, or the uterus may be stitched to the anterior abdominal wall through an abdominal opening, or it may be stitched forwards by an opening made by the vagina through the anterior fornix. In these cases trouble may arise if the woman should become pregnant, especially in fixations through the vagina. In cases of posterior displacement of the pregnant uterus retention of urine usually arises. The bladder becomes very much distended and fortunately an overflow of urine begins, or rupture would occur. This overflow is often mistaken for incontinence. The first thing to do in treating the case is to empty the bladder by catheter, but the whole of the urine should not be drawn off at once, or severe hæmorrhage may occur from the mucous membrane of the bladder. When the bladder is emptied the uterus should be replaced bimanually. The knee-elbow position will assist matters. The sound must not be used. In some cases the uterus is so jammed under the promontory that it cannot be released from below. The old method was to bring on an abortion, but it is better to open the abdomen and draw the uterus up. A pessary should be worn for a couple of months until the uterus has risen well above the brim of the pelvis.

### The Queen's Visit to the Louise Margaret Hospital, Aldershot.

BY OUR OWN CORRESPONDENT.

On Sunday afternoon the most casual observer passing by the Louise Margaret Hospital for Soldiers' Wives and Children at Aldershot must have seen that something unusual was afoot.

There was a note of preparation that could not be mistaken. Many people gathered together outside the gates, and among them were the proud parents of little ones inside, who passed round the news, "The Queen is coming.' As a matter of fact, it was not decided until the last moment that her Majesty would do more than draw up for a moment outside the gates. Inside the building the bright faces of the nurses passing quickly to and fro showed that they had no doubt whatever but that her Majesty would come.

The wards were prettily decorated with flowers, the children's ward looking especially charming, having been arranged entirely with marguerites, out of compliment to H.R.H. the Duchess of Connaught, whose name-flower it is. The only exception was a huge green jar on the centre table filled with magnificent scarlet poppies.

Fortunately there was no really serious case in the ward, and it was difficult to believe that the little ones looking so pretty in their scarlet and white jackets were "sick" at all.

Shortly before four o'clock the Duke and Duchess of Connaught, with Princess Margaret, drove up and were received by Lieut. M. G. Sterling, R.A.M.C., Medical Officer-in-Charge, and Miss C. Hodgson, the matron. Their Royal Highnesses are old friends to the hospital, as it was built during the Duke's command of the station, and was opened by the Duchess, receiving her names "Louise Margaret."

The Duke and Duchess went round all the wards, and the Duke was much amused by a "mother" in the maternity ward, in the confusion of showing off her three-days-old daughter, addressing him as "your Majesty." He laughingly replied that he was not his "Majesty," but his brother though! Their Royal Highnesses were much pleased to recognise in the children's or Queen Victoria ward Willie Kendall, a little fellow, now six years old, who was admitted three years ago suffering from hip disease. After having been two years on his back, he is now running about on crutches.

The Duke and Duchess had scarcely left the hospital when the Queen arrived, accompanied by the Prince and Princess of Wales and Princess Victoria. They were received by Lieutenant Sterling and Miss Hodgson, and visited all the wards, her Majesty speaking to each patient and smiling at each nurse as she passed round.

As the Royal party entered the children's ward the musical box struck up "God Save the King," and the Queen was much touched to see the little boy with hip disease leaning on his crutches and gravely saluting. Her Majesty turned to the Prince of Wales and said, "Look at the little darling saluting!"

A little girl about the same age also attracted much attention by saluting everyone who approached her chair with a "salaam," that is, dropping the head down into the hands, Hindoo fashion. The child (Gladys Richardson) has lived most of her short life in India, where she was paralysed by a sunstroke, and it was there she learned her quaint custom.

Her Majesty evinced much interest in all the nursing arrangements, asking many questions, and expressed herself heartily pleased with the maternity wards, which she thought well arranged for the comfort and well-being of the patients.

The Prince and Princess of Wales also asked many questions, showing, perhaps, the greatest interest in the children's ward. It was the first visit of the Commander-in-Chief, Lord Roberts, to the hospital, and the nursing staff were much pleased to see him there. Lord Roberts' love of children is well known, and he also conversed with little Willie Kendall, and expressed a hope that steps would be taken to ensure his future welfare when the time came for him to leave the hospital.

After visiting the wards the Queen asked to see the nurses' sitting-room, and there an amusing incident occurred. The hospital cat, a beautiful grey Persian, eight years old, fled before her Majesty and disappeared under the sofa. The Queen laughed and remarked, "Well, the cat does not like me at any rate," whereupon the matron apologised for its bad manners.

Before leaving the building, the whole of the Royal party signed their names in the "Visitors' Book," thus leaving a pleasant memento of a memorable day in the history of the hospital.

### The Nurses of the London Homœopathic Hospital.

A CHAT WITH THE MATRON. By OUR COMMISSIONER.

It speaks well for both the matron of the London Homœopathic Hospital and for her staff that many of the nurses have been associated with the institution for years, and have come to regard it in the light of their home. Miss Brew herself, with whom I had a chat the other day, after visiting the wards and the nurses' quarters, has been matron for more than a quarter of a century. It is, in fact, 27 years ago since she left the Royal Southern Hospital at Liverpool, to enter upon her duties in the old inconvenient building in Great Ormond Street, which in 1895 was replaced by one more suitable to present needs.

The matron candidly confessed that she does not attach great importance to the question of age. "We now admit probationers," she said, "at the age of 22, and if I thought a girl bright and capable who had not quite reached that age I should not shut the door against her. Neither, although, as a rule, it is better that anyone starting as a nurse should not be over 30, should I refuse a candidate who had passed her thirtieth year. A nurse came to this hospital when she had turned 30 and she has been here many years. On the other hand, one who came before she was 20 has been here 26 years."

"You raised the age of admission when the new hospital was opened?"

### The Primary Question.

"Yes; and of course we usually adhere to the regulation. The committee have all the papers submitted to them, and if I consider it desirable to recommend for admission a candidate under the prescribed age, they mostly assent to my recommendation. More important than the age is the personality of the woman, and as to the latter I am convinced that it is impossible to exercise too much care. Fitness for the work is the primary qualification."

"I conclude that the period of training is the same in all cases?"

"We make no exceptions whatever in that respect. Our period of training has always been three years, but in 1895 we added a fourth year of private nursing."

"With one month's trial?"

"No; candidates are only accepted after two months' trial. But if they are accepted, the payment dates from the time they enter."

### Salaries and Uniform.

"What is your scale of payment?"

"The probationers receive £8 the first year, £12 the second, £18 the third, and £25 the fourth, with board, lodging, washing, and indoor uniform."

"Are they expected to wear uniform out of doors?"

"I prefer it, but it is not always insisted upon. My opinion is that uniform is often a corrective for a little tawdriness of dress; and I like our nurses to look neat and trim always."

"Have you any difficulty in filling up vacancies?"

"There is always a number of suitable applicants waiting, and they come from Wales, Scotland, and Ireland, as well as from various parts of England."

### The Training.

"How often are the probationers examined during their training?"

"They receive elementary lectures on physiology and anatomy from the doctors, and are subject to examination in the instruction given during the first and second years. I do not give formal lectures, but I teach the probationers by personal direction. They come to me individually. A register of the progress made is kept, and is regularly placed before the committee, who are therefore in a position to know all that is going on."

### Duty and Diet.

"What are the hours of duty?"

"The day nurses are required to be in the wards at 7 A.M. They breakfast at 6.30. There is a short interval for lunch at 10. Dinner is at 12.30, and half an hour is allowed; also half an hour for tea. Supper is at 8, prayers at 9.45, bed at 10 P.M. The night nurses rise at 6.30 P.M., breakfast at 7.30. and are in the wards at 8. They have lunch at 1 A.M., and tea at 5 A.M. At 8.30 they go off duty and have supper. They are expected to be in their bedrooms by 11 A.M., and the bedrooms are darkened by noon."

"Have you any trouble about the diet for night nurses?"

"None at all. At one time there may have been a little difficulty, but since improvements were made at the opening of the new hospital, there have been no complaints. As a matter of fact, the night nurses now have as many meals, and quite as good fare as the day nurses."

"What time is allowed off duty?"

"Six hours every week. Every nurse is also permitted to be absent one day in the month from 10 A.M. to 10 P.M.; and for one half a day in the month. The annual holiday is three weeks."

### The Nurses' Home.

"I see that the nurses have very large sitting and dining rooms, but not separate bedrooms?"

"No, every dormitory has six beds, each of which is curtained off. Our space is restricted, and we have to do the best we can. The present nurses' home was the temporary hospital while the new building was in course of erection, and is connected with the permanent hospital by covered ways. The sisters have separate rooms."

"How many sisters are engaged in the hospital?"

"Six, and eight staff nurses. The remainder, making up from thirty-six to forty, according to requirements, are probationers in various stages of training."

### No Wardmaids.

"You do not have wardmaids?"

"They are not wanted. There are scrubbers who also clean the stoves, but the rest of the ward work is done by the probationers."

"Do any of them object to it?"

"I do not have any complaints. On the contrary, many take a pride in polishing the brasses and keeping everything in order. Those who have gone through the experience say it is most valuable. The nurses breakfast and dine in the house, but they have lunch and tea in the ward kitchen, which they could not do very well if we employed ward-maids. Apart from other reasons, there is no room for wardmaids."

### The Nursing Arrangements.

"What is the number of beds in each ward?"

"The larger wards contain fifteen beds, and the smaller nine. There is a large and a small ward on each floor. The number of beds is a hundred."

"And how many nurses?"

"A sister has charge of each floor, and there is a staff nurse in each ward. In the larger wards there are two probationers, and one in the small wards. There is a nurse in every ward during the night, whatever the nature of the cases; and one of the sisters has general charge at night."

"Then," continued the matron, "there is a sister, as well as a nurse, for the out-patients. I like all the nurses to get a turn in the out-patients' department. It is useful to them afterwards, especially if they take up district work."

### THE NURSES OF THE LONDON HOMŒOPATHIC HOSPITAL.—*Continued.*

THE PRIVATE STAFF.

"Do many of your nurses go in for district work?"

"A fair proportion. Five of our nurses went out to South Africa for the Army Nursing Reserve direct from the hospital, and three other members of the Army Nursing Reserve were trained here. Two of our nurses who returned with invalids came to see us. I am a little afraid that after the excitement of the work in South Africa nurses may find the work at home monotonous by comparison. As a rule our nurses remain with us."

"On the hospital staff?" "Either on the hospital or on the private staff. After their third year it is usual for them to serve on the private staff.

A NEW CO-OPERATION.

All the private nurses were trained here. They have a home in Queen's Square, and the home sister looks after them. Some of them have been with us for a long time. The hospital has the first claim on their services. I have two private nurses in the wards at the present moment. Perhaps you may like to know that we have just started a nurses' co-operation of our own."

"May I inquire why?"

"For the convenience of the doctors, and to keep the nurses connected with the hospital. At present there are only four; but it has only just begun. The nurses take their own fees, and pay 8 per cent. to the hospital."

At this stage I brought my inquiries to a close, but two or three other points of interest may be added. As I went through the wards, I learnt from a sister that the private nurses make all the curtains, the sheets, and the quilts for the children's ward; while the nurses in the isolation ward also make their own quilts. One ward of the hospital is constantly devoted to girls between 17 and 24, and to these special attention is given. The principle of caring for the stranger as well as that of maintaining the character of home, seems to be preserved throughout.

### Royal British Nurses' Association.

### ANNUAL MEETING.

THE annual meeting of the Royal British Nurses' Association was held at the Imperial Institute on Monday. In the absence of Princess Christian the chair was taken by Miss Thorold, who read the following telegram from Her Royal Highness:—"Quite unexpectedly unavoidably prevented presiding annual meeting to-day. Pray express my sincere regret to the meeting and my constant warm interest in the Association.—HELENA."

Miss Thorold then called upon Mr. John Langton, who gave a detailed explanation of the accounts. His announcement that Lily Duchess of Marlborough had promised her annual subscription of £100 was received with enthusiasm, and a unanimous vote of thanks to her Grace was passed. Dr. Alderson seconded the adoption of the financial statement, and a vote of thanks to Mr. Langton for his work as hon. treasurer was passed with applause.

Mr. Edward Fardon read the annual report from which it appeared that 147 nurses had applied for registration, of whom 132 had been accepted by the Board; that 134 new members had been elected, and 21 had withdrawn, principally on account of giving up nursing. Eleven members had died, among them being Miss Mary Shirley, lady superintendent of the Nurses' Institute at Stoke-on-Trent. Various members of the Association had had the honour of being mentioned in Lord Kitchener's despatches for honourable service during the war, and the distinction of the Royal Red Cross had been conferred on Miss Sidney J. Browne (acting matron of Queen Alexandra's Imperial Military Nursing Service), Miss E. A. Dowse, Miss L. M. Stewart, Miss A. Beadsmore Smith, Miss Blanch Trew, Miss Jessie Southwell, and Miss Edith Pretty. A substantial advance had been made in the subscriptions to the Helena Benevolent Fund, and a corresponding increase was shown in the grants made to members in sickness and distressed circumstances. Under the name of the Recluse Club an organisation had recently been formed to supply members who were ill or living alone, with weekly papers, etc., and a large number of nurses had already availed themselves of this privilege.

The report closed with the following paragraph:—

"The Midwives Bill was read a second time in the House of Commons on February 27th, and referred to the Grand Committee on Law. Efforts were made to secure amendments to the Bill, and a representation for this Association upon the Central Midwives' Board. These efforts were unsuccessful, but upon the Bill being reported on June 6th, an amendment to Clause 3 was agreed to by the House of Commons to the effect that one person should be appointed by the Royal British Nurses' Association. A special meeting of the Council was held on June 13th, and a resolution was agreed to that Clause 2 should be amended in such a way as to throw upon the Central Midwives' Board the whole responsibility of deciding who should receive certificates under the Act, and authorised the necessary steps to be taken to secure due consideration for such an amendment."

The report having been adopted, Miss Georgina Scott gave a few particulars about the Settlement Fund. The sum of £2,317 was now available for building, but over and above this, £100 a year would be required for maintenance. The treasurer had already opened an Endowment Fund, and the appeal had been most generously responded to. Nearly 50 promises of £1 a year had been given, and as the appeal was only issued early in June, this was very encouraging. Several nurses had promised this amount, and others, whose income was very limited while their expenses were heavy, had promised half-a-crown, five shillings, and other small sums. The Committee would be very glad of these, and would far rather have the £100 made up by small sums than by a large one, for while money could be counted up, the kindliness and sympathy which these small sums indicated could not be. "It is not the gift we look at," said Miss Scott, "but the giver."

The Council were re-elected, and the meeting closed with a vote of thanks to the President. Sir Dyce Duckworth, in proposing it, said he was sure that the Princess had some good reason for being absent, since all the members knew how the Association was in her heart, and how she showed herself one with them. The Association was full of life and vigour. The representation on the Midwives' Board was a matter for congratulation. There was no corporate body in the kingdom that had the same right, and whoever was appointed would be a source of strength to the Board.

This was seconded by Mrs. Dacre Craven, and carried with enthusiasm.

After lunch the members visited the exhibition at Earl's Court.

### THE MIDWIVES BILL.

Another special meeting of the Council of the Association was held on Friday last for the discussion of the proposed amendment to the Midwives Bill. The Chairman, Sir James Crichton Browne, said he understood that others in addition to Council members were present, but that they would not be allowed to take part in the discussion or to vote.

### ROYAL BRITISH NURSES' ASSOCIATION.—*Continued.*

The resolution before the meeting was as follows:—

That in order to secure that the full responsibility as to the admission of persons to the Midwives' Register should rest with the Central Midwives' Board, it is desirable that steps should be taken to procure an amendment to Clause 2 as it now stands by omitting lines 4 and 5.

Before it was entered upon a considerable amount of time was taken up in a discussion as to whether the conference agreed upon at the meeting of May 14th had been really held or not, and Dr. W. S. A. Griffith moved an amendment in which he submitted that the conference did not take place. Neither of the secretaries of the L.O.S., he said, had heard of it, and he proposed that, until a conference had been held, the Council should decline to proceed further in the matter.

Mr. Edward Fardon said he understood from the seconder of Dr. Griffith's resolution that only those members of the L.O.S. who were also members of the R.B.N.A. were to be invited to the conference. The amendment just proposed amounted to a vote of want of confidence in the Executive Committee.

Dr. Griffith differed as to the wording of his resolution. He said he was not asked to send it in in writing until a week after the meeting, and if he was right, the present meeting was invalid. Mr. Langton said the conference was held at his house. Four gentlemen and one lady were asked to attend. Two of the four were prevented. The names were: Sir John Williams, Dr. Duncan, Dr. Cullingworth, Dr. F. H. Champneys, and Miss Oldham, who represented the Midwives' Institute. The conference was a very amicable one, and he contended that it had been held.

Sir John Williams, who was present, said he had not the remotest idea that he was asked to that meeting as a representative of the L.O.S.

The Chairman said a conference did take place. If there had been any misunderstanding about the calling of it, that was past history. It might not have been regularly summoned; but if it had been held in the fullest manner, it could only report to this Council. It had made a verbal report to the Executive Committee, a meeting of which took place on the Friday preceding this Council meeting. A letter on the subject had since been received from Miss Wilson, president of the Midwives' Institute.

Mrs. Latter asked whether this might be read, but the request was not granted. The question, put by the gentleman who seconded Dr. Griffith's suggestion of a conference, "Have we any means of knowing what the opinions are of those who were represented at the conference?" was not replied to. Dr. Galton said there must be someone at the head of affairs who very strongly objected to the Bill, and he demurred to such meetings as the present one being held.

The amendment was lost, and after a lengthy discussion on the resolution, in which Sir John Williams, Dr. Griffith, Dr. Galton, Mrs. Latter, Miss Thorold, and a nurse-member took part, the resolution was carried, ten voting for it and six against. A number of ladies, the Chairman remarked, refrained from voting. Dr. Lesley Thorne, a member of the Executive Committee, said he had been in favour of the proposed amendments to the Bill, but that since hearing the remarks of Sir John Williams and Dr. Griffith he had altered his opinion.

### The Nurses' Hostel: Opening of the New Block.

BY A CORRESPONDENT.

The new premises of the Nurses' Hostel were opened on Monday, and a very large number of nurses and others interested in nursing matters attended. The South Block, like the original hostel, is situated in Francis Street, and at present it has been decided not to connect them by telephone, though probably this may come later on. The outside of the block is of warm red brick, the little bay windows, each with its blue and white willow pattern chintz curtains, being a pleasing feature. The secretary, Miss Paul, showed me all over the home, and I was especially impressed by the amount of light and air which permeated the whole establishment. The centre has a large well which is all lined round with white tiles, so that it may yield the largest possible amount of light, and here, in the narrow part of the building, are the bathrooms and the housemaids' closets for each floor. There are five floors, including the top one, which is partly occupied by the household staff; but there are also a few rooms for nurses at particularly low rates. The basement contains the nurses' dining-room—which runs the whole width of the house—a fairly large boxroom where boxes can be brought in from the outside through the courtyard without going into the house, a furnace-room for heating hot water, boot- and knife-cleaning rooms, a kitchen, scullery and pantry, and a small bedroom and sitting-room for the man and his wife who act as caretakers.

On the next floor is the nurses' sitting-room, a nice-sized apartment, with a grand piano, the sitting-room of the sister-in-charge, a waiting-room for general purposes, and several bedrooms. These are mostly reserved for occasional visitors, as it is found that regular inmates prefer the higher floors. The nurses may either rent an unfurnished room at £20 to £30 a year, or they may share any room above £26 a year with a friend, and so materially reduce the rent to be paid. Every room has a hanging cupboard in it, which is the property of the hostel. The nurses are allowed to sublet their rooms as long as references are satisfactory, but those who occupy the rooms have to pay 6d. a day for attendance to the management. The bedrooms with two beds, which are called cubicles, are provided, when let furnished, with two washstands and with a screen, which, like the curtains and the counterpanes, are of blue and white willow pattern chintz. There are 39 bedrooms in the house, exclusive of four for the staff. Of these half are let already. The management find that most of the nurses prefer to bring their own furniture, as it makes their rooms so much more homelike. One bedroom has a most noble-looking bed standing unusually high. The explanation was that the nurse had an extra foot in height put on to the legs of her bed—which were quite hidden by pretty draperies—so as to be able to keep her travelling trunks stored underneath out of the way. There is a gas range on the landing which will boil three kettles for a penny, or cook an egg or heat an iron, so that by combination only a third of a penny need be spent for the making of a special cup of tea. Baths cost one penny, boot-cleaning a halfpenny a pair, and breakfast can be had in bed for an extra 3d. For nurses passing through London bed and breakfast in a cubicle is 2s. only, a reasonable charge enough, and nurses who rent their own room pay 10s. 6d. only for board. In addition to a ladder giving exit to the roof, asbestos shoots which can be let down from the top floor to the ground will shortly be provided as fire escapes. All the unfurnished rooms in the hostel are being utilised for the crush of Coronation visitors by means of hired furniture.

### "The Hospital" Convalescent Fund.

The Hon. Secretary acknowledges with thanks the receipt of 2s. 6d. from each of the following nurses, for The Hospital Convalescent Fund:—Miss D'Arcy, Jarrow-on-Tyne Hospital and Miss H. M. Walker.

### Plaistow Maternity Charity and District Nurses' Home.

THE steady downpour of rain on the afternoon of Friday last augured ill for the attendance at the meeting of the Maternity Charity and District Nurses' Home, and it must have been all the more gratifying to Sister Katherine and the committee to see the large gathering that assembled, many supporters coming from some distance to Plaistow. Among the company were Viscountess Galway, Lady Lucy Hicks Beach, Lady Ebury, Lady Evelyn Ewart, Lady Albina Donaldson, the Hon. Jessie Lindley, the Bishops of Colchester and Barking, Mrs. Bonham Carter, Mr. Ernest Gray, M.P., and Canon Pelly. As the meeting opened a telegram was read from the Duchess of Sutherland expressing her great regret at being unable to take the chair as she had promised, on account of an order from the Earl Marshal to attend a rehearsal at Westminster Abbey. It was stated that her Grace was one of those deputed to carry the canopy over the Queen.

The Countess of Selborne then consented to take the chair on the condition that she should be allowed to *keep* the chair and not be expected to speak. The Bishop of Colchester therefore opened the proceedings, and referring to the splendid work done by the nurses during the past year said that they had received striking testimony from the medical officer of the Council of West Ham as to the great value of the services rendered during the recent outbreaks of enteric and small-pox. The Bishop appealed to his hearers to do their utmost to help this great work of charity and to interest their friends in it. If only people could be told about the value of the work, and the need it stood in of further funds, he was sure that they would not have to plead for help in vain. It was for this reason that it was decided to hold the meeting at the Home at Plaistow instead of at Stafford House, so that the West End might come to the East and judge for themselves, by a sight of the district, as to the reality of its needs. The Home was not in debt, but the committee had no working balance, and were in urgent need of money in hand.

Viscountess Galway bore testimony to the value of the work done by nurses trained at Plaistow in the rural districts. It was impossible, she said, to speak too highly of the admirable training the nurses received, especially in midwifery. She intimated that the Duchess of Sutherland had entrusted her with £5 to give to the funds of the Home, and had asked her to express her sincere regrets at her unavoidable absence.

Miss Amy Hughes, of Queen Victoria's Jubilee Institute for Nurses, said that she probably possessed a more intimate acquaintance with the work of the nurses both at Plaistow and in the country than anyone there present, because she had lived herself in the Home, and had seen the daily work of training the nurses, and had also visited many places in the country, and had seen the good work the nurses were carrying on all over England. The work that was done at Plaistow in training the nurses in midwifery would alone entitle the Home to the gratitude of the nation, and more especially to the dwellers in rural districts. It was in short the best school for training nurses that could be found, especially when it was considered how brief the time of training was, and how excellent the results obtained. It was indeed a pity that an institution that was at the head of all work of this kind should be crippled for want of funds. Miss Hughes added that it was at this Home that the Queen's Nurses received their obstetric training, and if it could obtain wider support it would become the national school for training nurses.

The Bishop of Barking, Mr. Ernest Gray, M.P., and the Mayor of West Ham paid tributes to the valuable services rendered by the nurses.

A short visit of inspection to the Home showed that the alterations which had been carried out during the past year were now complete; in fact, it was said that "the nurses now considered that they had everything they required." The baths on the basement floor with a splendid rush of water are a great boon. Whilst the tragedy of the last City fire is still fresh in the public mind, it was satisfactory to note the adequate precautions that are taken at the Home, including an outside iron staircase for use in case of need.

### Everybody's Opinion.

### A HOME FOR PRIVATE NURSES WANTED.

"A PRIVATE NURSE" writes: Will someone interested in nurses establish a home or boarding house where nurses who are engaged in private nursing can live comfortably and within their means? It is greatly needed, for no matter how economically one goes to work, living at St. Andrew's House means nearly £2 per week. Again, at the Hostel in Francis Street, no matter under what circumstance, you cannot have any food except at meal times. If you are going out early in the morning on a long journey, you go breakfastless; or you return from a long journey you have no food if it is not meal time. Such a place cannot be called a home; the block is well regulated and could be made cemfortable, but it is not home. A tired nurse, when she leaves her case, wants rest and a little comfort, not to share a room with two or three others as one has to do in houses provided by a certain association. A nurse must sacrifice herself very often and endure a great deal to get her patient well for her own credit and of her profession. She naturally requires some comfort and consideration when she is not at work. I am sure that a comfortable home would be a boon and a blessing, and would pay whoever had the courage to start it within easy distance of Oxford Circus.

### NURSES AND RELIGION.

"R. H. E." writes: A short time ago I wrote to the matron of a general hospital in London asking for an application form for probationer nurses. When I received it, I observed that, besides two ordinary references, a third was required, from a minister who knew the applicant. As I am not a member of any church, and do not attend regularly anywhere, I do not know a minister sufficiently to ask for a reference, and therefore I had to tell the matron that I could not give a "religious" reference; upon which I was informed that I could not be admitted to that hospital. The other day I went to see the matron of an infirmary in one of our large provincial towns, and almost the first question she asked was "What is your religion?" I said I had no creed, but I was not an atheist, and I did not desire to obtrude my views on others. The good lady replied that she considered character and religion went together, and further, that her hospital was quite unsectarian, but her nurses had to attend morning and afternoon services—Church of England—in the infirmary church every Sunday! I asked if, as attendance at a place of worship was compulsory, and I preferred Nonconformist services, I should be allowed to attend outside churches in place of the infirmary one. "No, the rule could not be altered," the matron said, and went on to tell me that amongst her nurses were Unitarians, and many others belonging to different denominations, who all attended the Church of England services, and made no objection to doing so. I wondered if their religion were either not very real to them or if they were compelled to give up the liberty of their conscience to earn their living. A wrong in either case. Finally I was told that it would be no good for me to apply. I have now written to another hospital, but the same questions are asked, and if I answer them truthfully, I shall probably never get a chance of being a nurse in this, or any other hospital which, I think, is decidedly unjust. If a candidate gives the ordinary references, and they are satisfactory, I think that should be sufficient, and certainly she ought not to be obliged to refer to a clergyman or minister, or compelled to attend certain churches or chapels. For I fail to see why a woman of good moral character, and with the necessary qualifications, should be debarred from taking up the nursing profession because she does not possess a conventional religion, and is too honest to pretend to have what she has not. Surely it is time that questions about religion, on application forms for nurses, should be omitted.

### Echoes from the Outside World.

### Movements of Royalty.

On Saturday evening the King and Queen left London for Aldershot. Upon their arrival at six o'clock they received an address from the Urban District Council, and the Queen was presented with a bouquet. As the Royal visitors passed through the town the school children in Wellington Avenue sang the National Anthem and then broke out into childish and delighted applause, and a loyal populace lined the streets all the way from the station to the Pavilion where their Majesties stayed. This is the only military residence of the Sovereign, and it is a low one-storeyed building, practically designed by the late Prince Consort, standing in spacious and well-wooded grounds. At ten o'clock there was a torchlight tattoo, which would have been remarkably enjoyable if the weather had been warm and balmy. English, Welsh, Scottish, and Irish soldiers took part in the ceremony, and the effect of the flaring lights, the steady tramp of the men, and the music of the massed bands, was most striking. The ceremony was over soon after eleven, but unfortunately, owing to the inclemency of the weather, the King contracted a chill and was unable to be present at Divine Service on the Sunday morning, which was attended by the Queen and a number of members of the Royal family On Monday, the Prince of Wales took his father's place at the Review, and Queen Alexandra presented the new colours to the Highland Light Infantry. About 32,000 soldiers were present at the Review. In the afternoon the King was sufficiently recovered to proceed to Windsor. He did not go to Ascot on Tuesday, but went for a drive in the Long Walk in a closed carriage. The Queen drove in State to the races, accompanied by several members of the Royal Family.

### Inspection by the Prince of Wales.

Twelve thousand members of Brigades for Boys assembled on the Horse Guards' Parade on Saturday afternoon, and were inspected by the Prince of Wales. The downpour of the morning was so persistent that there had been some talk of postponing the function, but the bitter disappointment shown by the lads, some of whom had come from Ireland and Scotland for the event—and had been camping in the open behind Lambeth Palace—decided the officers to let matters take their course, and fortunately the weather cleared somewhat after two o'clock. There were eight battalions of the Boys' Brigade, numbering 4,000; eight of the Church Lads' Brigade, also 4,000; four battalions of the London Diocesan Church Lads' Brigade, 2,000; and one battalion of the Catholic Boys' Brigade, 800. The latter had six field guns as well as little hand wagons. On the right of the parade ground the various bands were massed, and in the background could be seen the field hospital of 100 beds, which had been established in St. James's Park for the treatment of casualties. At the windows of the Levée Room of the Horse Guards the boys were rejoiced to perceive the Princess of Wales, accompanied by Prince Edward, Prince Albert, and the little Princess Mary. Prince Edward was evidently delighted at the march past of the lads, some of whom were not much older than himself, and occasionally clapped his hands with vigour. The Prince of Wales wore the uniform of a Colonel of the Royal Fusiliers, and rode a magnificent black charger. He inspected the various battalions, and then the boys marched past. The military officers present expressed themselves surprised at the precision of the movements and the smart appearance of the boys.

### Hats at the Coronation.

The inconvenience of the matinée hat to those around has often been demonstrated, but this is the first occasion when the matter has been considered of sufficient importance to be mentioned in Parliament. Last week Mr. Cremer asked the First Commissioner "whether ladies occupying seats upon the stands in Palace Yard on the 26th and 27th inst. will do so subject to the rational conditions upon which they are admitted to the Coronation ceremonies in the Abbey; or if ladies are to be permitted to retain their headdress during the passage of the Royal cortège, whether he will make it a condition that the covering of ladies' heads shall be of such limited proportions and so arranged that members seated behind them may have an opportunity of seeing the procession, instead of having their view interrupted by ladies hats." The First Commissioner of Works replied that it did not fall within his province to issue such regulations, and no conditions have been laid down. "Matters of this kind," he added, "must, I think, be left to the good feeling of those concerned." Although the authorities are unable to take any active steps in the direction desired, it is quite possible that the mere fact of the subject having been brought before the House of Commons may cause ladies who would have erred from want of thought to reconsider the matter, and order a hat of dainty proportions. If they could be brought to seriously believe that unless their head gear is of reasonable size they will be called upon by indignant seat-holders to remove it as the procession files by, the concession asked for would be made without further trouble.

### The Boer Surrenders Completed.

The Boer surrenders are now complete, and amount to 17,700. General De Wet, who during the war was such a brave foe, seems determined to be a loyal friend and to use all the influence he possesses to promote loyalty among others. Addressing an enormous assembly of Boers nine miles from Winburg he said: "Perhaps it is hard for you to hear from my mouth the announcement that we have a new Government, but God has decided thus, and we were obliged to part with our cause, which we had upheld for two years and eight months. As a Christian people, God now demands us to be faithful to our new Government." On Tuesday Lord Kitchener reported to the War Office that the South African Constabulary had been handed over to the civil authorities, the necessity for any further military operations having now ceased.

### Foreign.

The new Prime Minister of France, M. Combes, is 67 years of age, and a many sided man. Educated at a Roman Catholic seminary, he took orders early in life, but he has since entered the medical profession, and has achieved eminence as a doctor. He is also a specialist on education. His Cabinet contains several new men, and his programme comprises the reduction of the period of military service to two years; the application in its spirit as well as in the letter of the Association Law; the abrogation of the Falloux Law, and the establishment of a general income tax. The new Budget is to be one of severe economy, "in which the figures will not conceal the truth from the taxpayer."

### Art.

Last Saturday was a memorable day at Christie's salerooms. A portrait of Miss Sarah Rodbard, painted by Romney in 1784, and exhibited at the British Institute in 1855, was put up to auction. It is a large canvas, and though some critics were heard to remark that they considered the picture had been a little overcleaned, and was not by any means the finest of Romney's productions, it was ultimately purchased by Mr. Locket Agnew for 10,500 guineas. Strangely enough this was the exact sum paid in 1896 for another portrait by Romney, "The Viscountess Clifden and Lady Spencer." Ten pictures given by a lady to be sold for the benefit of the Manchester Infirmary fetched the handsome sum of 1,069 guineas, and altogether the prices realised were eminently satisfactory.

An exhibition of 30 cabinet pictures by Mr. Byam Shaw at Dowdeswell's Gallery in New Bond Street is particularly interesting. It it entitled, "Sermons in Stones and Good in Everything," and the subjects of the various pictures have been suggested by the Book of Ecclesiastes. One illustration will serve to show the tone taken by the artist. The text chosen is "But the abundance of the rich will not suffer him to sleep." The picture is that of a modern wealthy householder, attired in dressing-gown and slippers, armed with a revolver and carrying a candle, searching for suspected burglars in his suburban residence. Mr. Shaw does not confine himself to any particular period in his illustrations, but includes Puritans, Florentines, Cavaliers, etc., and all are treated with the ability which distinguishes this young artist.

### Appointments.

[No charge is made for announcements under this head, and we are always glad to receive, and publish, appointments. But it is essential that in all cases the school of training should be given.]

Barrington's Hospital, Limerick.—Miss Ellen O. Coffey has been appointed matron. She was trained for four years at Sir P. Dun's Hospital, Dublin, where she was afterwards on the private nursing staff for five years. She has since, for nine months, been staff nurse at the Barrington Hospital, Limerick.

Border Counties Home for Incurables, Carlisle.—Miss Florence M. Huelin has been appointed matron. She was trained at Leeds General Infirmary, and has since been matron at the Cottage Hospital, Whitby.

Colchester Hospital.—Miss Annie Nutter has been appointed night sister. She was trained at Leeds Infirmary for three years. She has since been charge nurse at Prescot Infirmary, and staff nurse at West Ham Hospital.

County Hospital, Newport, Mon.—Miss L. T. Evans has been appointed sister. She was trained for four years at the London Hospital, and has since been sister at the General Hospital, Worcester.

Jarrow-on-Tyne Hospital. — Miss d'Arcy has been appointed matron. She was trained at the Sussex County Hospital, and has been sister, Aberdeen Royal Infirmary; night superintendent, Worcester General Infirmary, and night superintendent, Wolverhampton and Staffordshire General Hospital; lady superintendent, Andover Cottage Hospital; matron, Malmesbury Hospital; and matron, Bridgend Hospital.

Newcastle-upon-Tyne Lying-in Hospital.—Mrs. Mary Jane Sloane has been appointed matron. She was trained at the Rotunda Hospital, Dublin, where she has since been in succession staff nurse, night superintendent, and sister-in-charge of a portion of the maternity department.

South Charitable Infirmary, Cork.—Miss Alice M. Bleakley has been appointed charge nurse of the Protestant wards. She was trained at the General Hospital, Tunbridge Wells.

Taunton Isolation Hospital.—Miss Mary MacKay has been appointed nurse-matron. She was trained at Knightswood Small-pox Hospital, Glasgow, of which she has since had entire charge. She has also been sister of the scarlet fever Pavilion at Middleward Hospital, Motherwell, and assistant matron at Nitshill Fever Hospital.

Watford Isolation Hospital.—Miss Fanny Fairbrother has been appointed sister. She was trained at Hampstead General Hospital for three years, and has since been nurse at Beacon Hill Hospital, Faversham. She has also done private nursing, and been attached to a nursing home at Southport.

### Presentations.

Hants County Hospital.—Miss Amy Bullock, who is leaving the Royal Hants County Hospital to take up new work as first lady superintendent of the Nurses' Hostel at Horsham, has been presented with the following tokens of regard:—A framed etching of Worcester Cathedral from the matron, a copy of the latest edition of "Christian Year" from the chaplain, a travelling clock from the nursing staff, and a silver tea set from the porters and maids.

Woodlands Convalescent Home, Rawdon. — The Ladies Committee of the Woodlands Convalescent Home, Rawdon, on Tuesday, presented Miss Miller, the matron, who has resigned her post, with a timepiece and gold bracelet, as a mark of their appreciation of her valuable work at the home for more than nine years, and expressed their deep regret at the loss of her services to the home.

### For Reading to the Sick.

"NOT DEATH, BUT LIFE."

Oh! this is not to die—
To leave a world of changes and of seeming,
Where amid fleeting phantasies we dwell;
And wing away, as from a state of dreaming,
To waking and to Bliss unchangeable.

Oh! this is not to die—
Is it not rather into Life expanding,
Breaking the trial-state to live indeed?
Safe from the tempest in the haven landing,
From storms, from toils, from rocking billows freed?

No! no! we cannot die—
In Death's unrobing room, we strip from round us
The garments of mortality and earth;
And breaking from the embryo state which bound us,
Our day of dying is our day of birth!

And yet to earth we die—
Born to new Life with all its weight of Blessing,
Born to a world where ills can never press;
Exalted, pure, Angelic joys possessing,
If this be Death, then Death is Happiness!

*Dean Newman*

Two things underlie man's dread of what awaits him beyond death—his ignorance of the future state and his sense of sin. Jesus meets both these needs. He meets our dread of the future as of an unknown state, by giving us a revelation of the state of the faithful departed, "Thou shalt be with me in Paradise." Hence we learn that the death of a penitent believer in Jesus Christ is a birth into a more glorious condition of life. Our death is like the death of Jesus Christ. As He in His death was quickened in spirit, and passed into a state of glorious power, so our death is an entrance into the same life.

A material body is not a necessary condition of man's life. He is not an animal, but a spirit dwelling for a while in an animal organism. Just as Jesus' personality is in His divine nature, so man's personality is in his spiritual nature

Death is no passing into a state of unconsciousness or inactivity. I know that in the Bible the dead are spoken of as "them which are asleep." But this term is used where death is considered as it acts on the body. We are taught to believe, not only in the immortality of the soul, but the resurrection of the body; and here death is but a sleep; the grave of a loved one is but a bed where angels keep their watch over the sleeping bodies of the faithful; and when the morning breaks, as at His second advent, the Sun of Righteousness arises on this world; the sleepers, aroused by His voice, shall awake and come forth. "All that are in the graves shall hear His voice, and shall come forth." Fittingly, therefore, is the term "sleep" applied to the repose of the bodies of the faithful in the grave.

But they sleep not in their spirits. Does the spirit ever sleep, even here? Truly in Paradise they rest; but the rest of spirits is like the Sabbath of God, a rest of restful activity. No oblivion enfolds them in an intense sleep, for in them consciousness lives on "Thou shalt be with me in Paradise." This fact underlies all the New Testament revelations of the state of departed Christians.

*Canon Body.*

### Notes and Queries.

**The Editor is always willing to answer in this column, without any fee, all reasonable questions, as soon as possible.**
**But the following rules must be carefully observed:—**
1. **Every communication must be accompanied by the name and address of the writer.**
2. **The question must always bear upon nursing, directly or indirectly.**

**If an answer is required by letter a fee of half-a-crown must be enclosed with the note containing the inquiry, and we cannot undertake to forward letters addressed to correspondents making inquiries. It is therefore requested that our readers will not enclose either a stamp or a stamped envelope.**

*Tuberculosis of Throat.*

(87) Can you tell me where a banker's clerk, age 28, suffering from tuberculosis of throat and of one lung, can be taken in for treatment, either free or for a nominal fee? He has spent his all at Nordrach, and in a voyage to New Zealand.—*M. B. C.*

You might apply to the Hospital for Consumption, Brompton, S.W., or the North London Hospital for Consumption, Mount Vernon, Hampstead, N.W.

*Maternity.*

(88) Can you tell me of an institute where I could be trained in maternity work without paying a premium?—*Nurse Muriel.*

If you are a trained nurse some private home might be willing to train you on reciprocal terms. You might advertise.

1. Will you kindly tell me where it is possible to get midwifery training in return for services, or for a very small fee? 2. Can you advise as to the best way of getting permanent cases or other in London for a nurse with nearly two years' training and some private experience in Edinburgh, and who, owing to rheumatism, has been unable to finish her full course of training?—*Scotia.*

1. You may hear of a private home which will give you the instruction which you require by advertisement. Could you not call upon the matron of the Edinburgh Royal Maternity Hospital, 79 Lauriston Place? The fees are not heavy, and perhaps to a trained nurse she might be able to offer special terms. 2. Your best plan is to advertise. As the nurse suffers from rheumatism, it might be advantageous to her to obtain work at one of the hospitals or homes at one of the mineral water-baths.

I am desirous of going out to India as a maternity nurse. I hold the L.O.S. and British Lying-in Hospital certificates; and I have been told by a doctor in the Indian Medical Service that there is a good opening in India for nurses of that kind who are gentlewomen, and that I should apply to the India Office. Will you kindly tell me where I should apply, and to whom?—*F. S.*

The India Office is concerned with military nursing and not with maternity work. You might join one of the associations of private nursing, if you are sufficiently qualified in general nursing to do so. It is of the utmost importance, especially in districts remote from medical aid, that maternity nurses should hold high qualifications in the general as well as the special branches of their calling.

Can you tell me where I could obtain "Examination Questions" which have been set by the London Obstetrical Society?—*B. B.*

Apply to the Secretary of the Society, 20 Hanover Square, London.

*Uniform.*

(89) A nurse attached to a private nursing home of which the uniform is a dark-blue cloak, and bonnet would be glad to know if it would be breaking nursing rules if she wore a veil to her bonnet. It would be a convenience to her to have a veil as her hair is short. *Nurse Mary.*

You had better obtain permission from the lady superintendent of the home to which you are attached.

*Pharmacy.*

(90) Can you tell me of any hospital where a lady can receive training in dispensing which will enable her to take the Apothecaries' Hall qualification? Could such a pupil reside in the hospital?—*Florence.*

Write to the Secretary, the Women's Medical School, Handel Street, W.C.

*Address.*

(91) Will you kindly tell me the address of the Colonial Nursing Association in Scotland?—*S. H. K.*

The address of the Colonial Nursing Association is the Imperial Institute, London, S.W.

*Book.*

(92) Will you kindly tell me the best book to study for the diseases of children, their treatment and nursing?—*D. C. N.*

"The Mother's Help to the Domestic Management of her Children," by P. Murray Braidwood, M.D., is very good. "Treatment" and "nursing" are, however, widely different subjects, and are hardly likely to be dealt with fully in the same book.

*Brass Plate.*

(93) My wife wants a brass name-plate; will you kindly suggest the proper place to put the "L.O.S." Should it be "Mrs. S——, L.O.S. Certificated Midwife," or "Mrs. S——, Certificated Midwife, L.O.S. Diploma"?—*W. J.*

Why not "Mrs. S——, Midwife, L.O.S. Certificate"?

*Ice Poultices.*

(94) 1. Will you kindly tell me the best way to make an ice poultice? 2. Will you also say why surgical poultices should be made on tow and medical ones on poultice cloth?—*Bootle.*

1. Use an ice-bag. 2. Surgical poultices are made on tow in order that they may be burnt when done with. Medical poultices are often made on tow for the same reason.

*Open-air Treatment.*

(95) I should be greatly obliged if you could tell me of any institution where a child of nine could be received for the open-air treatment, free, or for a small payment?—*Nurse R.*

The Mount Vernon Hospital for Consumption, Hampstead, N.W.

*Hospital Training.*

(96) I am anxious to train for nursing in a children's hospital, but I am near sighted and very short. Do you think that I should be accepted?—*P. O.*

Probably not, but you can do no harm in applying.

Which special hospital gives the best training in nursing women's diseases?—*C. F. S.*

The Chelsea Hospital for Women, Fulham Road, S.W., and the Hospital for Women, Soho Square, W., both afford good training.

Will you kindly tell me if a three years' training in a workhouse hospital is enough preparation for an Army nurse, or if a certificate from a general hospital is required?—*U. W. S.*

Candidates for the Army Nursing Service must hold a three years' certificate from a civil general hospital.

Will you kindly tell me if a three years' certificate obtained from a large union infirmary, which is a recognised training school, is considered on the same level as one obtained from an infirmary not under the Local Government Board? Will the above certificate allow a nurse to hold a post as sister in any hospital or infirmary?—*M. S. M.*

A nurse must obtain a three years' certificate from a training school recognised by the Local Government Board, to enable her to become superintendent nurse in any of the Poor Law infirmaries. The subordinate positions may be filled by nurses who have had different qualifications at the discretion of the hospital authorities.

Will you kindly tell me if there is any hospital which will accept a woman of 42, either as paying probationer or otherwise?—*M. E. H.*

See the "Nursing Profession: How and Where to Train." There are one or two schools which possibly might accept an exceptionally suitable candicate of that age as paying probationer.

*Royal British Nurses' Association.*

(97) I should be obliged if you would give me particulars o the Royal British Nurses' Association, as I should like to become a member of it.—*P. E. K.*

Apply to the Secretary, 10 Orchard Street, W.

*Swedish Medical Movements.*

(98) Will you please tell me if there is any institution in London where the Swedish medical movements can be learnt?—*Nurse.*

Apply to the Hon. Secretary, the British College of Physical Education, Lancaster Gate, London, W.

*Engadine.*

(99) I have been nursing during this season in San Remo, and should like to continue working through one summer in Switzerland or the Engadine. Can you give me the names of any nursing institutes where trained nurses are sent out to private patients?—*L. M.*

Perhaps the Davos Invalids' Home, Davos Dorf, Switzerland, would suit you.

*Deafness.*

(100) Will you kindly tell me of a hospital in London where by paying a small fee I could get advice for deafness? I could afford to pay a little but not to go to a specialist.—*K. T.*

You can be treated at the Central London Throat, Nose, and Ear Hospital, Gray's Inn Road, W.C., or at the London Throat Hospital, 204 Great Portland Street, W., either free or by the payment of a small fee.

**Standard Books of Reference.**

**"The Nursing Profession: How and Where to Train." 2s. net; post free 2s. 4d.**
**"Burdett's Official Nursing Directory." 3s. net; post free, 3s. 4d.**
**"Burdett's Hospitals and Charities." 5s.**
**"The Nurses' Dictionary of Medical Terms." 2s.**
**"Burdett's Series of Nursing Text-Books." 1s. each.**
**"A Handbook for Nurses." (Illustrated). 5s.**
**"Nursing: Its Theory and Practice." New Edition. 3s. 6d.**
**"Helps in Sickness and to Health." Fifteenth Thousand. 5s.**
**"The Physiological Feeding of Infants." 1s.**
**"The Physiological Nursery Chart." 1s.; post free, 1s. 3d.**
**"Hospital Expenditure: The Commissariat." 2s. 6d.**

**All these are published by the SCIENTIFIC PRESS, Ltd., and may be obtained through any bookseller or direct from the publishers, 28 and 29 Southampton Street, London, W.C.**

